# Selective visual attention to drive cognitive brain–machine interfaces: from concepts to neurofeedback and rehabilitation applications

**DOI:** 10.3389/fnsys.2014.00144

**Published:** 2014-08-12

**Authors:** Elaine Astrand, Claire Wardak, Suliann Ben Hamed

**Affiliations:** CNRS, Cognitive Neuroscience Center, UMR 5229, University of Lyon 1Bron Cedex, France

**Keywords:** brain–machine interfaces, brain–computer interfaces, cognition, spatial attention, neurofeedback, neural training, frontal eye field, prefrontal cortex

## Abstract

Brain–machine interfaces (BMIs) using motor cortical activity to drive an external effector like a screen cursor or a robotic arm have seen enormous success and proven their great rehabilitation potential. An emerging parallel effort is now directed to BMIs controlled by endogenous cognitive activity, also called cognitive BMIs. While more challenging, this approach opens new dimensions to the rehabilitation of cognitive disorders. In the present work, we focus on BMIs driven by visuospatial attention signals and we provide a critical review of these studies in the light of the accumulated knowledge about the psychophysics, anatomy, and neurophysiology of visual spatial attention. Importantly, we provide a unique comparative overview of the several studies, ranging from non-invasive to invasive human and non-human primates studies, that decode attention-related information from ongoing neuronal activity. We discuss these studies in the light of the challenges attention-driven cognitive BMIs have to face. In a second part of the review, we discuss past and current attention-based neurofeedback studies, describing both the covert effects of neurofeedback onto neuronal activity and its overt behavioral effects. Importantly, we compare neurofeedback studies based on the amplitude of cortical activity to studies based on the enhancement of cortical information content. Last, we discuss several lines of future research and applications for attention-driven cognitive brain-computer interfaces (BCIs), including the rehabilitation of cognitive deficits, restored communication in locked-in patients, and open-field applications for enhanced cognition in normal subjects. The core motivation of this work is the key idea that the improvement of current cognitive BMIs for therapeutic and open field applications needs to be grounded in a proper interdisciplinary understanding of the physiology of the cognitive function of interest, be it spatial attention, working memory or any other cognitive signal.

## INTRODUCTION

Only a couple of decades ago imagining an interface between the human brain and a machine was more of a science fiction than of a scientific endeavor. Chapin et al. (1999) pioneered the field with the first demonstration of a real time motor brain–machine interface (BMI) that is the demonstration that brain activity from the rat motor cortex can be used to control a robotic arm. This achievement brought about the realization of the enormous potential of the field, which up to this day has not ceased to expand.

The main objective of motor BMIs is the rehabilitation of patients with major motor deficits yet preserved cortical motor functions. Applications involve for example controlling a screen cursor using motor or premotor cortex brain activity in monkeys (Serruya et al., 2002; Taylor et al., 2002; Santhanam et al., 2006; Kim et al., 2011) and in tetraplegic human patients (Hochberg et al., 2006) which has been proven feasible with a remarkable spatial accuracy. As computer-assisted aids continue to permeate our everyday life environments, this approach is expected to grant patients who have difficulties moving their arms or hands an increased autonomy and freedom of action. Pushing BMIs yet a step further, several studies show that motor cortical activities can also be used to control robotic arms in their reaching and grasping components with an impressive degree of precision, both in monkeys (Carmena et al., 2003; Tillery et al., 2003; Lebedev et al., 2005; Velliste et al., 2008; Ifft et al., 2013) and in humans. This has for example allowed a tetraplegic patient to help herself with a drink with the aid of an artificial arm controlled by her motor cortex activity (Hochberg et al., 2012).

Several new directions are currently being explored by BMI research. For example, a recent study demonstrates that incorporating sensory feedback to a motor brain-computer interface (BCI) improves its performance (Suminski et al., 2010). Along another line, Shanechi et al. (2014) demonstrate that the signals decoded from the motor cortical activity recorded in a monkey performing a sensorimotor task can be used to stimulate the spinal cord and muscles of a second anesthetized monkey giving rise to directed movements of its limb toward distinct targets. This study opens new rehabilitation perspectives for paralyzed patients. The exhaustive review of the major advances in motor BMIs and their novel perspectives is however beyond the scope of the present review.

## BASIC PRINCIPLES UNDERLYING BRAIN–MACHINE INTERFACES

**FIGURE 1:**
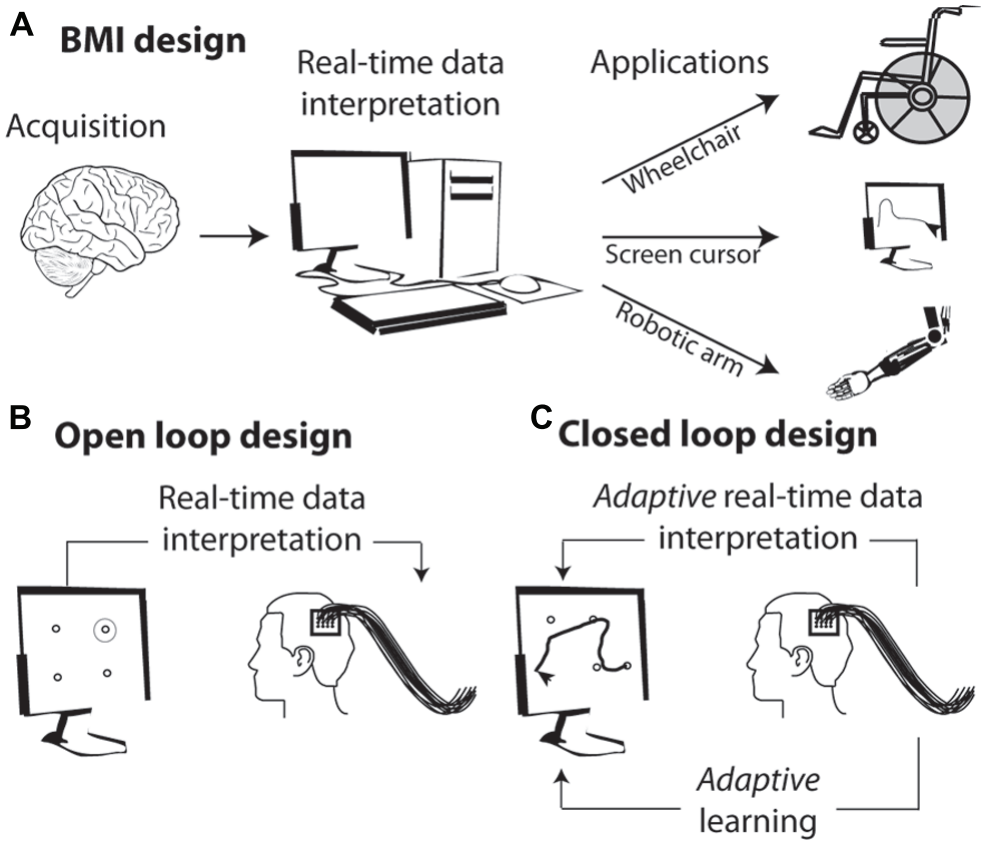
**From brain signals to controlled external effectors. (A)** Schematic representation of the workings behind brain–machine interfaces (BMI). **(B)** Open loop BMI. The subject is here implanted with intracortical electrodes in the primary motor cortex. The activity of this cortical region is used to select a target on the screen without the subject being required to adjust his or her neuronal activity. **(C)** Closed loop design. The activity of the motor cortex is used to guide a cursor on the screen. The subject sees the screen cursor in real time and is required to adapt his or her brain activity in order to increase the precision of the cursor’s trajectory.

The basic concept behind BMIs is the interpretation, in real time, of cortical neuronal population activities and their translation into a goal directed action through a diversity of external effectors (**Figure 1A**). Developing a BMI includes two distinct phases (**Figure 1B**): (1) a learning or training phase during which a classifier learns to associate the observed instantaneous simultaneous activity of a neuronal population with the actual state of the variable of interest (the position of a stimulus in space, the direction of an intended motor plan, the spatial location of visuospatial attention, or the content of short term memory etc.); (2) a testing phase during which the classifier defined in the learning phase is used to define the most probable state of the variable of interest, given the observed instantaneous simultaneous activity of the recorded neuronal population. Above-chance decoding accuracy indicates that the neuronal population contains reliable information about the variable of interest.

In its simplest form, the mapping between the neuronal code and the desired output relies on the interpretation of the subject’s endogenous neuronal codes. For example identifying the neuronal codes with which a set of movements is encoded in the motor cortex allows to associate a given neuronal population activity (e.g., corresponding to moving one’s arm left) with a specific spatially congruent external effector output (e.g., moving a cursor left, **Figure 1B**). More complex designs are further based on the adaptive capabilities of the primate cortex and rely on learning and positive reinforcement procedures. In these designs, subjects learn to produce the neuronal population activities that best control the effector output, thanks to a sensory feedback (e.g., seeing the cursor’s trajectory) that allows them to assess how well they are successful at controlling the external effector (**Figure 1C**).

## COGNITIVE BRAIN–MACHINE INTERFACES (cBMI)

While most of the research effort in neural prosthetics has concentrated on the use of motor signals to drive external devices, new directions in the field of BMIs are also emerging. For example, Musallam et al. (2004) have shown, in the context of motor behavior, that cognitive signals such as the expected value of a reward, i.e., the subject’s motivation, can be decoded, at the single trial time scale, from parietal neural activity. Jerbi et al. (2009) show that such signals as attention orientation signals and mental calculation signals can be used to drive a cognitive BCI. Instead of decoding movement-related signals from motor specific cortical activity, these cognitive BMIs (cBMIs) seek to access the content of cognitive processes. One of their principal long-term goals is to develop therapeutic tools for the treatment of cognitive disorders.

**FIGURE 2:**
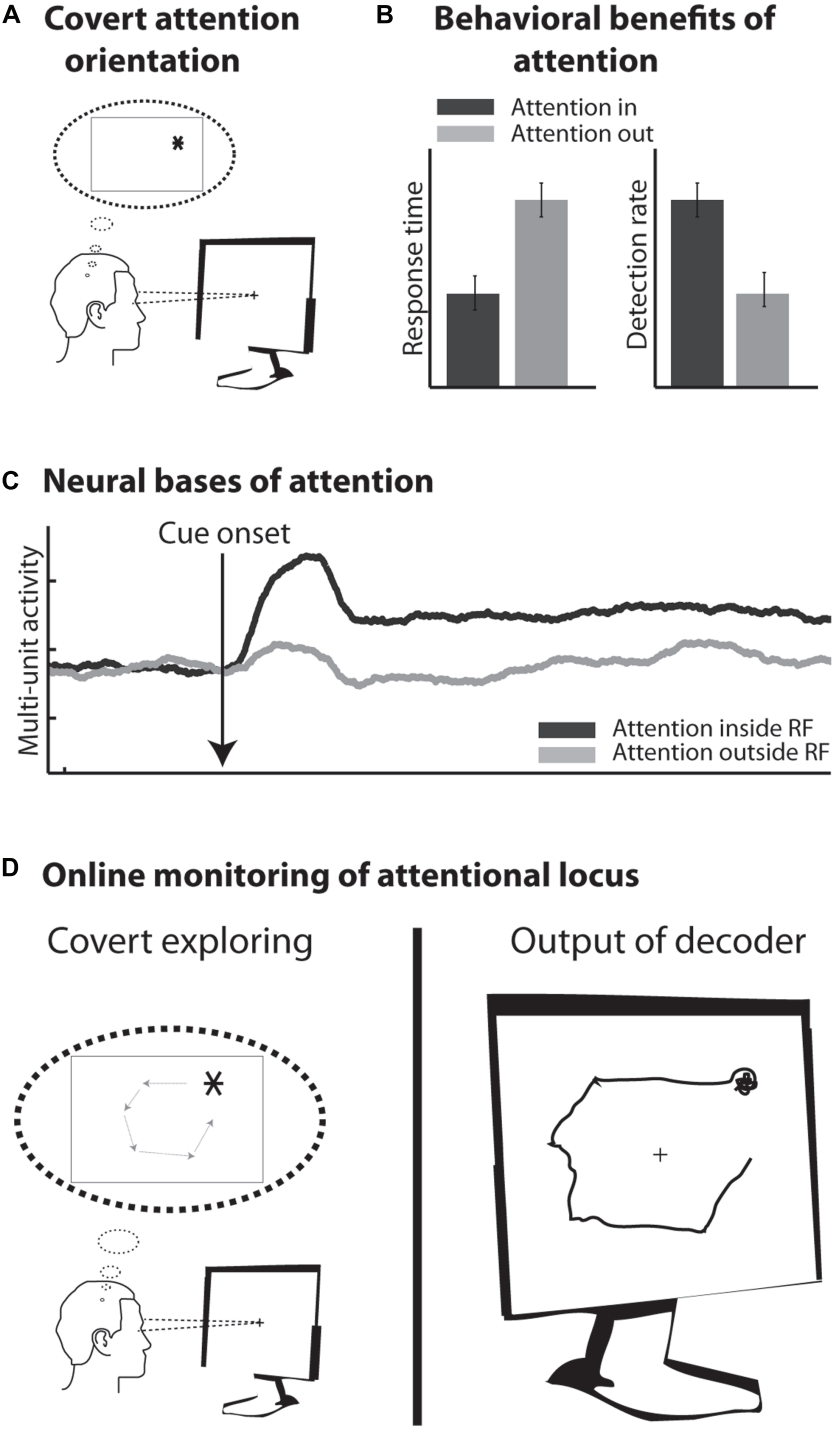
**(A)** Covert orienting of attention involves selecting a peripheral location in space (here *) while the eyes remain at the center of the screen. **(B)** Attention orientation results in faster response times and higher detection rates in response to visual stimuli presented at the attended location, as compared to the non-attended location. **(C)** Attention orientation enhances both the phasic and tonic neuronal response components in several cortical areas including the frontal eye fields (FEF). Schematic representation of this effect onto multi-unit neuronal activity. **(D)** Online monitoring of the spatial locus of attention. On the left: the subject is covertly moving his attention to covertly explore the screen. On the right: the decoder interprets in real time the activity related to the subject’s attention orientation.

In healthy subjects, motor cortical commands can be objectified and time-locked to overt limb displacements. In contrast, cognitive processes, such as planning, holding information in short-term memory or orienting one’s attention in the environment, are essentially internal subjective processes. From a behavioral point of view, their content is covert and can be inferred only indirectly from their effects on other overt measures (e.g., oral report, reaction time measures, detection rates etc.). From a neurophysiological point of view, their neural bases are increasingly understood and clear neuronal signatures can be assigned to them. However, unlike sensory or motor processes, these cognitive processes cannot be precisely time-locked to objective external events. In addition, they are often multiplexed with sensory, motor as well as other cognitive signals. As a result, cBMIs currently appear as more challenging than motor BMIs. The present review focuses on a major cognitive function, namely visuospatial attention (**Figure 2A**), which is known to enhance visual processing both at the behavioral (**Figure 2B**) and neurophysiological levels (**Figure 2C**, see below). It proposes a precisely quantified comparative overview of the different cBMI approaches that have been developed to decode this cognitive signal at the scale of the single trial (**Figure 2D**). It also explores the initial steps at using such cBMIs for cognitive rehabilitation purposes. These studies are discussed in the light of the accumulated knowledge about the psychophysics, anatomy, and neurophysiology of visual spatial attention. The core motivation of the present review is the key idea that the improvement of current cBMIs for therapeutic and open field applications needs to be grounded in a proper interdisciplinary understanding of the physiology of the cognitive function of interest, be it spatial attention, working memory or any other cognitive signal.

## VISUOSPATIAL ATTENTION AND ITS NEURAL CORRELATES

Orienting one’s attention toward a given location in space enhances visual processing at that location (**Figure 2B**). Reaction times are faster (Posner et al., 1980, but see also Albares et al., 2011), spatial processing (Bashinski and Bacharach, 1980; Prinzmetal et al., 2005; Ibos et al., 2009) and spatial resolution are enhanced at the attended location (Yeshurun and Carrasco, 1998; Gobell and Carrasco, 2005; Carrasco and Yeshurun, 2009; Anton-Erxleben and Carrasco, 2013) and spatial representation is distorted up to several degrees away from the attended location (Wardak et al., 2011a). At the neuronal level, attention is described to modulate both the baseline (e.g., Armstrong et al., 2009; Ibos et al., 2013) and the visual responses (e.g., McAdams and Maunsell, 1999), to decrease neuronal response latency (Lee et al., 2007), as well as to modify the neurons’ spatial selectivity profiles (Ben Hamed et al., 1997, 2002; Anton-Erxleben et al., 2009). At the neuronal population level, attention is also thought to decrease interneuronal correlations (Cohen and Maunsell, 2009).

Spatial orienting of attention can be achieved through two different mechanisms. It can either be driven by external stimuli that capture attention. This mechanism is referred to as exogenous, bottom-up or involuntary attention. Alternatively, attention can be voluntarily driven by internal goals. This mechanism is referred to as endogenous, top-down or voluntary attention. Early on, Posner et al. (1980) and Jonides (1981) suggested that a single cortical system controls both the endogenous and the exogenous orientation of attention. In contrast with this proposal, Müller and Rabbitt (1989) postulated that the endogenous and exogenous orienting of attention are functionally distinct and constitute separate mechanisms in constant competition with each other (e.g., Zénon et al., 2008, 2009). Confirming this view, recent functional magnetic resonance imaging (fMRI)-studies demonstrate the co-existence of two distinct frontoparietal networks involved in orienting attention (Corbetta and Shulman, 2002): a dorsal network that is active during top-down attentional control, i.e., when attention is internally maintained or voluntarily driven (Kastner et al., 1999; Shulman et al., 1999; Corbetta et al., 2000; Hopfinger et al., 2000; Kincade et al., 2005), and a ventral network that is activated when attention is reoriented both voluntarily and by relevant but unexpected stimuli (Arrington et al., 2000; Corbetta et al., 2000; Macaluso et al., 2002; Kincade et al., 2005; Vossel et al., 2006). In the non-human primate, a bilateral frontoparietal attentional network involving the frontal eye fields (FEF; Bruce and Goldberg, 1985) and the lateral intraparietal area (LIP; Barash et al., 1991; Ben Hamed et al., 2001) is described. These areas are activated by both endogenously driven attention and exogenously driven attention to task-relevant stimuli (Gottlieb et al., 1998; Armstrong et al., 2009; Gregoriou et al., 2009; Suzuki and Gottlieb, 2013). Interestingly, during endogenous top-down driven attention, FEF neurons tend to respond earlier than LIP neurons whereas during exogenous bottom-up driven attention, the inverse is observed (Buschman and Miller, 2007; Ibos et al., 2013). The distinction between a dorsal and a ventral frontoparietal network is still unclear in the macaque monkey. The FEF possibly belongs to a putative monkey dorsal attentional network while area 45, ventral to area FEF, possibly belongs to a putative monkey ventral attentional network (Wardak et al., 2011b).

## VISUOSPATIAL ATTENTIONAL SIGNALS FROM A cBMI PERSPECTIVE

### TIME-LOCKING

The feasibility of cBMIs based on visuospatial attention signals as compared to motor or sensory BMIs depends on the existence of neuronal population activity patterns that distinguish between whether the subject is orienting its attention say to the left or to the right of the visual field. The P300 speller is an example of a successful non-invasive cBMI driven by the decoding of visuospatial attentional locus as inferred from the modulation of neuronal responses to a visual stimulus presented at the specific locus of attention. Another approach is to decode sustained attentional brain correlates toward different spatial positions from neuronal population activities. This approach is different because decoding such sustained information cannot be precisely time-locked to an external event. As a result, identifying its precise neuronal activation pattern during the training phase of the decoding is in itself a challenge.

### DECODING ATTENTION-RELATED CORTICAL INFORMATION

One important question is whether such attentional information, because of its self-initiated endogenous nature, can be decoded with an accuracy that is comparable to that obtained from sensory and motor related activities. Two studies are interesting in this respect. Armstrong et al. (2009) recorded neuronal responses from the macaque FEF while the animals were involved in a cued target detection task. The cue was an exogenous cue, having the same spatial location as the target. Astrand et al. (2014a) also recorded neuronal responses from the macaque FEF while the animals were involved in a cued target detection task, except that in this case, the cue was calling for an endogenous orienting of attention toward the cued location (FarbodKia et al., 2011; Astrand et al., 2014a). An exogenous cue is thought to involuntarily shift the subject’s attention to its location whereas to shift attention to a location indicated by an endogenous cue, the subject needs to voluntarily orient its attention toward it (Jonides, 1981). Both studies thus manipulate spatial attention but the cue that was used called for different orientation processes. Armstrong et al. (2009) show that both visual and sustained attention information can be decoded from a macaque FEF population of neurons modulated both by visual stimuli and attention orientation. Specifically, they report 100% accuracy for the decoding of the spatial location of a visual peripheral stimulus and up to 90% accuracy for the decoding of the spatial locus of sustained attention. They also describe a lower decoding variability for a visual stimulus than for sustained attention. Astrand et al. (2014a) report a similar trend on a random neuronal population selection including both attention-selective neurons and attention non-selective neurons. They further quantify the sensitivity of decoding accuracy to neuronal population size and trial number. In addition, they show that, using populations of visual-selective and attention-related neurons of the exact same size, comparable decoding accuracies are obtained for both variables. This is a strong indication that both exogenous (visual) and endogenous (attention-related) information are encoded in the cortex with a similar reliability. As a result, cognitive variables such as attention can be expected to be decoded from neuronal population activities with similar accuracies as those achieved when decoding sensory or motor variables (for example, see Ben Hamed et al., 2003, 2007).

## USING ATTENTION-RELATED SIGNALS TO CONTROL A BRAIN–MACHINE INTERFACE

**FIGURE 3:**
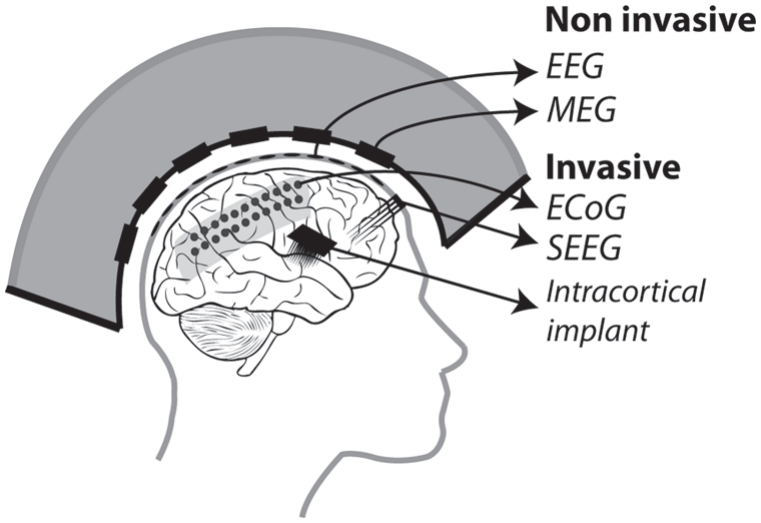
**Different recording methods used to control BMIs.** Invasive methods includes: ECoG electrodes placed on the dura, SEEG electrodes placed through the skull into the cortex and intracortical electrodes implanted in the cortex. Non-invasive methods include: EEG electrodes placed on the scalp and MEG squids placed around the head.

In the following, we will present an overview of recent studies evaluating the accuracy with which attention-related cortical information can be decoded. These range from non-invasive recording studies in humans [MagnetoEncephaloGraphic recordings (MEG), Electroencephalographic (EEG) recordings and fMRI] to invasive recording studies in human (ECoG) and non-human primate [ECoG, multi-unit neuronal activity (MUA) recordings and single-unit neuronal activity (SUA) recordings] subjects (**Figure 3**). Whenever possible, we document for each study the following information (**Table 1**): (1) the type of signal component each study relies on, (2), whether attention is driven endogenously or exogenously, (3) how well each method succeeds at decoding attentional engagement signals (i.e., the fact that the subject is focusing its attention as compared to no attentional focus), (4) how well each method allows to distinguish between left and right attentional orientation, (5) how well each method allows to distinguish between attentional loci situated in the same visual hemifield, and (6) how visual distractors interfere with the decoding of attention. Points 4 and 5 reflect the spatial resolution with which attention-related signals can be accessed. All this comparative information is presented in **Table 1**. Importantly, to be considered as robust, it has been proposed that BCI performance needs to be above 70% (Kübler et al., 2004, 2006), a threshold accuracy to keep in mind while analyzing **Table 1**.

**Table 1:** Comparative overview of the non-invasive EEG and MEG and invasive ECoG, SEEG and neuronal recording studies, in humans and non-human primates, that have aimed at decoding attention-related information from the ongoing neuronal activity in a perspective of using these signals to drive BMIs.

	Study	Signal	Attention	Atentional engagement	Average decoding performance	Left/right attention	Up/down attention	Distracters
Non-invasive	van Gerven and Jensen (2009)	MEG/Alpha power (8–14 Hz) occipito-parietal channels	Endogenous	–	*2 positions*: 69% (*n* = 15 subjects, chance at 50%) *4 positions*: 41% (*n* = 15 subjects, chance at 25%)	Five best subjects: 78%	Five best subjects: 58%	–
	Treder et al. (2011)	EEG/Alpha power	Endogenous	–	*2 positions*: 75% (*n* = 8 subjects, chance at 50%)	–	–	–
	Morioka et al. (2014)	EEG/Alpha and Beta Power guided by NIRS	*Exogenous*	–	*2 positions*: 79% (*n* = 8 subjects, chance at 50%)	79%	–	–
	Andersson et al. (2011)	fMRI/7T	Endogenous	–	–	*Online fixed threshold*:** L: 41%/R: 25% (*n* = 7) *Offline fixed threshold*: L: 89%/R: 88% (*n* = 7) *Online adaptive threshold*: L: 75%/R: 85% (*n* = 2)	–	–
	Andersson et al. (2012)	fMRI	Endogenous	–	*2 positions*: 79% (*n* = 9 subjects, chance at 50%)	77%	82%	–
*Invasive in humans*	Gunduz et al. (2012)	ECoG/Power from all frequency bands	Endogenous	84% (chance 50%)	*3 positions*: 48% (35% during motor engagement) (chance at 33%)	–	–	–
	Andersson et al. (2011)	ECoG/High gamma power	Endogenous	–	*3 positions*: 70% (chance at 33%)	Left: 55%/Right: 60%/Center: 82%	–	–
*Invasive in non-human primates*	Rotermund et al. (2013)	ECoG/Power, phase coherence and difference	*Exogenous*	–	*2 positions*: 96% (chance at 50%)	94%	99%	Present but not quantified
	Astrand et al. (2014a)	SUA/average spiking rate	Endogenous	–	–	82%	–	Present but not quantified
	Astrand et al. (2014b)	MUA/average spiking rate	Endogenous/*Exogenous*	90–100%	*4 positions*: 67%/79% (chance at 25%)	–	–	–
	Armstrong et al. (2009)	SUA/average spiking rate	*Exogenous*	–	*2 positions*: 90% (chance at 50%)	–	–	20% drop in performance
	Zhang et al. (2011)	SUA/average spiking rate	Endogenous	–	*3 positions*: 56% (chance at 33%)	–	*3 positions* 56%	10% drop in performance

### NON-INVASIVE STUDIES IN HUMANS (MEG, EEG, AND fMRI)

The first attempt at decoding attention orientation at the single trial level is that of van Gerven and Jensen (2009). The authors recorded the cortical activity using MEG while subjects were covertly attending to one amongst four possible locations in space, previously indicated by an endogenous central cue. Analyzing these signals oﬄine, and specifically the alpha-band power (8–14 Hz) of the parieto-occipital captors, they report an average decoding performance of 69% when discriminating between two possible spatial locations (chance being at 50%). They observe better decoding performances at discriminating *left/right* locations (78%, average calculated over their best subjects) than *up/down* locations (58%, *ibid*). They additionally report an average decoding performance of 41% when discriminating between four possible spatial locations (chance being at 25%). This study thus provided the first evidence that endogenous spatial orientation of attention can potentially be monitored in real time. A subsequent EEG study, using a very similar experimental design reports an average performance of 75% (chance being at 50%) based on the alpha power of the recorded signals (Treder et al., 2011). Morioka et al. (2014), report a slightly higher average decoding performance (79%) using near-infrared spectroscopy (NIRS) as prior information for the analysis of the EEG signals. It is however not clear if this improved performance results from using the NIRS prior information or is due to the fact that an exogenous cue was used instead of an endogenous cue. Using fMRI, Andersson et al. (2011, 2012) take the field one step further by decoding, in real time, the spatial orientation of attention between three endogenously cued locations (one central and two peripheral locations, 2011) and five endogenously cued locations (one central and four peripheral locations, 2012).When using an adaptive threshold on the classification of the real-time BOLD signal, thanks to a real-time design, they obtain a decoding performance of attention orientation (79, 75% at decoding attention to the left and 85% at decoding attention to the right) equal to that obtained with EEG. In contrast with van Gerven and Jensen (2009), they demonstrate a slightly better performance at discriminating between up/down attention (82%) than between left/right attention (77%). When reprocessed oﬄine the average decoding performances reported by Andersson et al. (2011, 2012) reaches 88% (89% at decoding attention to the left and 88% at decoding attention to the right).This sets the ground for a promising future in the field of attention cBMI. Importantly, it is to be noted that all of these non-invasive studies aiming at decoding spatial attention signals from human cortical activities report an important intersubject variability, overall attention orientation decoding performance being high in some subjects and almost at chance in other subjects. See **Table 1** for comparison of the above studies.

### INVASIVE STUDIES IN HUMANS (ECoG)

Drawing nearer to the source of the cortical signals, ECoG studies in patients implanted for clinical purposes predicted higher performances for decoding spatial attention than non-invasive techniques. Gunduz et al. (2012) report a performance of 84% at decoding whether a subject is engaging its spatial attention or not using the spectral power amplitude from all frequency bands and a performance of only 48% at decoding the location of attention amongst three possible spatial positions (chance at 33%). Andersson et al. (2011) focus on the high gamma power and report an average of performance of 70% in a similar design (chance at 33%). The discrepancy between these two studies most probably reflects the dependence of the decoding performance upon the exact localization of the ECoG electrode arrays in each subject. Interestingly, Andersson et al. (2011) used the exact same design to decode spatial attention both from ECoG implanted patients and from subjects included in the fMRI experiment described in the previous section. Surprisingly enough, the average decoding performance is higher in the fMRI-based non-invasive approach than in the ECoG implanted patient. This is most probably due to the fact that the fMRI-based protocol specifically relies on the analysis of the regions of interest (ROIs) that are specifically activated by attention orientation, while the ECoG recording correspond to an averaged smoothed analog of these signals. See **Table 1** for comparison of the above studies.

### INVASIVE STUDIES IN NON-HUMAN PRIMATES (ECoG, SUA, MUA)

These approaches are expected to yield the highest decoding performances as compared to the two previous approaches, due to the fact that the recording are specifically targeted to the cortical regions involved in attention processing (closest to these regions, on the cortical surface for ECoG, and right within these regions for MUA and SUA recordings), though the number of simultaneously recorded signal sources is also a variable to take into account (the higher the number of recording contacts, the higher the expected decoding performance). MUA can be considered as averaged SUA, as MUA represents several neurons at the same time while SUA represents well identified individual neurons. In a very elegant study, Rotermund et al. (2013) demonstrate extremely high accuracies in discriminating between two exogenously driven attentional loci (average 96%), with slightly better performance for two attentional loci situated in the same visual hemifield (99%) as compared to two attentional loci situated in different visual hemifields (94%), in agreement with the non-invasive fMRI driven study by Andersson et al. (2012). Importantly, Rotermund et al. (2013) placed the ECoG array onto the visual areas V1 and V4 the activity of which is known to be strongly modulated by attention, according to a strict topography matching attention allocation. While these areas can functionally be considered as downstream from the parietofrontal network described above, the fact that attention can be decoded with such high performances from these regions is noteworthy given that these areas are not expected to be subjected to eye-movement or motor planning interferences that can possibly degrade the decoding of spatial attention in other cortical regions (see below). This performance is to be compared with the performance of 90% at predicting the location of attentional locus amongst two possible locations (inside or outside the receptive field) following an endogenous cue (Armstrong et al., 2009), when using the SUA of attention-selective cells recorded in the monkey prefrontal cortex (FEF). Astrand et al. (2014a) report that it is possible to predict whether a monkey was orienting its attention to the right or to the left visual field from the activity of a cortical population of mixed selectivities (attentional and non-attentional) with a performance of 82%. Both these studies (Armstrong et al., 2009; Astrand et al., 2014a) correspond to a coarse approximation of real-time decoding of attention orientation, as they involve the artificial concatenation of cells recorded in independent sessions and having different spatial selectivities. More recently, Astrand et al. (2014b) report, in a real-time decoding design, a 67% performance at discriminating between four possible attentional locations from MUA recordings in the FEF, following an endogenous cue (79% when attention is oriented exogenously). The performance with which they can predict whether the monkey has engaged its attention or not is around 90% for endogenous cueing (98% when attention is oriented exogenously).

### DEPENDENCE OF DECODING PERFORMANCE ON THE EXPERIMENTAL DESIGN

Classification accuracy increases as a function of the number of recorded signals and as a function of the number of available training trials (e.g., Armstrong et al., 2009; Astrand et al., 2014a). The impact of these two parameters on classification accuracy depends on the classification algorithm being used. Indeed, regularized linear regression classifiers appear to be more resilient to low number of recorded signals and low number of trials than other decoders (e.g., support vector machine, reservoir, linear regression, see Astrand et al., 2014a). The impact of these two parameters on classification accuracy also depends on how much information about the variable of interest is present in each recorded signal. For example, a 75% decoding performance of spatial attention can be achieved with as few as 15 trials per condition when each recorded signal has been selected on the basis of its high attention-related content (e.g., attention-related cells in SUA decoding studies). When no prior selection is exerted onto the recorded signals, up to 80 trials are required to reach this 75% decoding performance (Astrand et al., 2014a). As the number of recorded signals and/or the number of trials increases, the impact of further increasing any of these two parameters onto the decoding performance decreases (Astrand et al., 2014a). **Table 2** summarizes, for each of the different studies decoding spatial attention discussed above, the number of recorded signals and how they are placed with respect to the brain, the classifier being used as well as the number of available trials for the classification analysis. In general, there appears to be a trade-off between the number of recorded signals and the number of trials required to achieve a high decoding performance (Astrand et al., 2014a). A precise quantification of this trade-off per type of recorded signal, from non-invasive to invasive, is unfortunately missing and would be extremely useful. This would allow a more direct comparison between the different types of studies. But most importantly, this would allow to better adjust the experimental design to the constraints of the method being used. One expects for example that a higher number of trials will significantly improve the decoding performance achieved in implanted patients. Quantifying this trade-off is all the more important if we want to move from a two-class decoding design (e.g., left/right) to a multi-class (e.g., upper left/upper right/lower right/lower left) or to a 2D continuous decoding design (e.g., x, y), in which case, the number of available trials per condition clearly becomes a limitative parameter.

**Table 2:** Comparative overview of the type of signals used for the decoding of spatial attention in the studies discussed in **Table 1**.

	Study	Signal	# Recorded signals	Coverage	Classification	Available trials
**Non-invasive**	van Gerven and Jensen (2009)	MEG/Alpha power (8–14 Hz) occipito-parietal channels	275 DC Squid axial gradiometers	Full head	Support Vector Machine w. linear kernel	128 trials per condition
	Treder et al. (2011)	EEG/Alpha power	64 channels	Full head	Logistic regression with L2 regularizer	40 trials per position
	Morioka et al. (2014)	EEG/Alpha and Beta Power guided by NIRS	EEG: 64 channels. NIRS: 49 channels.	Full head EEG. Parietal and occipital NIRS	Sparse logistic regression	88 trials per position
	Andersson et al. (2011)	fMRI/7T	Two ROIs: right vs. left and left vs. right	Full head		8 trials (13s) per condition
	Andersson et al. (2012)	fMRI		Full head		9 trials (13s) per position
**Invasive in humans**	Gunduz et al. (2012)	ECoG/Power from all frequency bands	86, 78, 64, 103, and 88 electrodes	Left hemisphere, 1 subject right hemisphere. No occipital lobe	Stepwise regression and Bayesian classification	40 trials per position (except for one subject: 80)
	Andersson et al. (2011)	ECoG/High gamma power	64 electrodes	Left parietal-occipital cortex		20 left/20 right/39 center
**Invasive in non-human primates**	Rotermund et al. (2013)	ECoG/Power, phase coherence and difference	36 and 37 electrodes	Extrastriate area V4 and portions of V1/V2 along lunate sulcus	Support Vector Machine w. Gaussian kernel	~several hundred trials
	Astrand et al. (2014a)	SUA/average spiking rate	131 neurons	Single hemisphere, Right or Left Frontal Eye Fields	Linear Regression with regularization	60 trials per condition
	Astrand et al. (2014b)	MUA/average spiking rate	48 channels	Both hemispheres, Frontal Eye Fields	Linear Regression with regularization	60 trials per position
	Armstrong et al. (2009)	SUA/average spiking rate	40 neurons	Right Frontal Eye Field	Support Vector Machine w. linear kernel	$>$10 trials per condition (leave-one out procedure)
	Zhang et al. (2011)	SUA/average spiking rate	187 neurons	Anterior Inferior Temporal cortex, single hemisphere	Correlation Coefficient Classifier	12 trials per stimulus (leave-one-out procedure)

*This includes the type of recording method, the number of recorded signals, brain coverage, the type of classifier used for the decoding, the available number of trials per condition.*

Overall, most of these studies report a decoding performance above the 70% criteria for a robust BMI (Kübler et al., 2004, 2006). However, in spite of the fact that several experimental parameters contribute to the final decoding performance, invasive attention-based approaches in the non-human primate produce the highest decoding performances as compared to both invasive and non-invasive recording approaches in humans. This provides grounds of improvement for the latter. This is most probably due to the fact that the recordings can be performed closest to the source of the attention-related signals. Supporting this hypothesis, fMRI decoding of spatial attention (driven by activations in ROIs specifically identified based on their contribution to spatial attention processes) outperforms all other non-invasive decoding approaches.

## CHALLENGES OF ATTENTION-DRIVEN COGNITIVE BRAIN–MACHINE INTERFACES

### ENDOGENOUS VERSUS EXOGENOUS ATTENTION

As seen above, attention can be voluntarily controlled by the subject or involuntarily oriented by an external event. An efficient cBMI is expected to be able to infer spatial attention signals independently of how attention has been driven. Given the functionally partially distinct neural bases of endogenous and exogenous attention orientation, it is thus crucial to quantify, from a cBMI perspective, (1) whether both types of attention orientation modes lead to comparable decoding accuracies, i.e., whether the performance of a cBMI driven by endogenous attention signals is comparable to that of a cBMI driven by exogenous attention signals; and (2) whether a cBMI driven by say endogenous attention signals can generalize and also be driven by exogenous attention signals and vice versa. As discussed above, a recent study shows that the decoding of spatial attention during a cued target detection task from MUA recordings in the non-human primate FEF is partially dependent on whether attention is oriented endogenously or exogenously (Astrand et al., 2014b). Importantly, the decoding performance of exogenous attention signals (79%) is higher than that obtained when decoding endogenous attention signals (67%). Most interestingly, they additionally show that a classifier trained at decoding endogenous attention successfully reads out exogenous spatial attention neuronal signals (54%), though significantly less than if the classifier is directly trained on exogenous attention (drop of 13%). The relationship is not symmetrical as a classifier trained at decoding exogenous attention successfully reads out endogenous spatial attention neuronal signals with a performance of 62%, again slightly lower than if the classifier is directly trained on endogenous attention (drop of 5%).

### SUSCEPTIBILITY OF ATTENTION-DRIVEN COGNITIVE BRAIN–MACHINE INTERFACES TO SENSORY AND COGNITIVE FACTORS

Neuronal signals collected from the primary motor cortex are only marginally affected by changes in the sensory environment (e.g., changes in the visual or somatosensory information) or by changes in the cognitive context (e.g., changes in what the subject is thinking about or planning to do). As a result, the generalization capabilities of motor BMIs are unaffected by such varying circumstances. It is still unclear whether this is also the case for cBMIs. While the several studies cited above quantified how well a cognitive variable, namely spatial attention, can be predicted from cortical activity response patterns, none directly tested whether and how this prediction was affected by either a change in the sensory environment or in the cognitive context. A recent report by Astrand et al. (2013) shows that the decoding of spatial attention orientation during the delay period of a memory-guided saccade task is affected by the presence of visual noise. Precisely, they report a 62% performance at decoding spatial attention orientation and spatial short-term memory information from MUA activities recorded in the non-human primate FEF during the delay period of a memory-guided saccade task in the absence of any visual noise and a performance of 63% in the presence of visual noise. When a classifier is trained at decoding this spatial attention orientation in the absence of visual noise and is tested on MUA activities collected in the presence of visual noise (or vice versa) performance drops by 12%, though it remains well above chance. The decoding of spatial attention signals are thus affected by changes in the sensory environment. Astrand et al. (2013) similarly show that the decoding of the spatial position of a visual stimulus from MUA activities recorded in the non-human primate FEF depends on whether the monkey is for example performing a memory-guided saccade task or a simple target detection task (decoding performance across contexts leads to a drop in 18%, Astrand et al., 2013). Last, as described above, the authors also show that the decoding performance of spatial attention depends on whether attention is oriented endogenously or exogenously, a situation that can be seen as a change in the cognitive context (Astrand et al., 2014b).

### SUSCEPTIBILITY OF ATTENTION-DRIVEN COGNITIVE BRAIN–MACHINE INTERFACES TO DISTRACTERS

Another important aspect to consider is how distracters affect the spatial location of attention and hence the stability of a cBMI based on spatial attention signals. Such distracters can be considered as a specific case of a sensory change in the subject’s environment (cf. above).In an urban environment where we are constantly exposed to salient stimuli, the endogenous attention that corresponds to an internal objective that the subject wishes to achieve will be continually interrupted by a diversity of external stimuli, both relevant (traffic lights, car horns, your children’s voice etc.) or irrelevant (christmas lights, traffic flow, strangers passing by etc.).Several behavioral studies show that peripheral cues automatically capture attention (Jonides and Irwing, 1981; Christ and Abrams, 2006; Neo and Chua, 2006; Schreij et al., 2008). However, attentional capture is not constant. For example, when subjects are explicitly instructed to ignore a peripheral cue, attentional capture is reduced, though not completely abolished (Lambert et al., 1987). Similarly, if attention is highly focused, for example when the subject strongly expects a visual event at a certain location (e.g., following a 100% validity cue), the attentional capture as measured from reaction times is extremely weak (Yantis and Jonides, 1990). In contrast, if the subject is expecting a visual event that can take place at an undefined location (its attention is thus diffuse, e.g., following a 25% validity cue), the attentional capture is much stronger (Yantis and Jonides, 1990). The same reduction in attentional capture can also be observed in visual search experiments where subjects need to distribute their attention over the whole search display in order to scan the scene efficiently (Schreij et al., 2008). Last, attentional capture also depends on the complexity of the visual scene (which is the case of our everyday environment). Cosman and Vecera (2009) show that, when subjects need to search for an item in a complex environment, attentional capture declines as the complexity of the visual scene increases.

These behavioral observations are in agreement with single cell recordings in the monkey parietal cortex. For example, the neurons of the LIP (Ben Hamed et al., 2001), an area functionally associated with attentional processes (Wardak et al., 2002, 2004), are specifically activated by behaviorally relevant visual events independently of whether relevance is due to the intrinsic properties of the stimuli (e.g., an abrupt onset high contrast stimulus) or to its extrinsic properties (e.g., a low contrast stimulus, the processing of which is important to the ongoing task, Gottlieb et al., 1998; Kusunoki et al., 2000). Spatial attentional priority is suggested to be encoded by the differential response between the neurons encoding a specific spatial location against the response of the entire LIP population (Bisley and Goldberg, 2003). Consequently, the selection of a spatial location by attention can be biased by focal LIP optogenetic or electrical microstimulation, mimicking an attention interference or capture (Dai et al., 2014). A recent study by Suzuki and Gottlieb (2013) further suggests that these suppression mechanisms might differ between the prefrontal and parietal nodes of the parietofrontal attentional network. Specifically, the neuronal response to distractors is weaker in the prefrontal cortex than in parietal cortex, indicating a stronger suppression. Additionally, the degree of this suppression with behavioral suppression markers is stronger in the prefrontal cortex. Last, reversible inactivation of the prefrontal cortex leads to a more severe distractability than observed following inactivation of the parietal cortex.

From a decoding perspective, Astrand et al. (2014b) show that a distracter interferes with the performance with which spatial attention can be decoded from FEF MUA recordings in the non-human primates performing a cued target detection task. As observed by others (Armstrong et al., 2009; Zhang et al., 2011), distractors interfere with the accuracy with which spatial attention can be decoded on correct trials. Astrand et al. (2014b) further show that this interference is maximal on false alarm trials, i.e., on trials in which the monkey erroneously responded to the distractor instead of waiting for the target. The distractor interference is similar between correct trials and trials on which the monkey missed the target. Remarkably, the accuracy with which spatial attention can be decoded is much lower on incorrect trials than on correct trials, whether attention has been oriented endogenously (25%) or exogenously (40%), a trend also reported by Armstrong et al. (2009).

### SUSCEPTIBILITY OF ATTENTION-DRIVEN COGNITIVE BRAIN–MACHINE INTERFACES TO EYE MOVEMENTS

The last constraint that needs to be discussed in the context of attention-driven BMIs is eye movements. Indeed, the attentional frontoparietal network described above is highly overlapping with, though distinct from, the cortical oculomotor network (Corbetta et al., 1998; Wardak et al., 2006). In all of the studies considered above, the subjects are required to maintain eye fixation during the decoding procedure. As a result, they are behaviorally constrained to suppress an oculomotor-related signal. Studies evaluating the impact of eye movements on cBMIs are yet missing. Treder et al. (2011) demonstrate that high accuracy for EEG-based classification is often associated with low accuracy for eye movement electrooculography (EOG)-based classification, and vice versa. This suggests a dissociation between EEG- and EOG-based classification. It also indicates that eye movements disturb the decoding of attention orientation if not taken into account. Gunduz et al. (2012) further show that, in the absence of any prior processing, the performance with which attention orientation can be decoded in ECoG-implanted patients drops from 48 to 35% when the subjects are planning their motor response (i.e., hardly above the 33% chance level). This suggests that a “naïve” attention decoding performance is most probably disrupted by other signals than just eye-movements, including motor planning. The exact location of the recording sensors is expected to highly impact on such interferences. However, generally speaking, real-time denoising algorithms minimizing the impact of eye movement signals over the attention-related signals are potentially promising. Extremely simple strategies such as analyzing cortical signals only at a distance from eye movements might also prove sufficient.

Overall, in spite of the notable progress in the field of attention-driven cBMIs, several challenges need to be faced before large field therapeutical applications can be considered. A tight interaction between the field of real-time decoding of cortical activity and cognitive neurosciences is expected to have a major impact on facing these challenges, the growing understanding of how the brain functions playing a crucial role toward refined machine learning strategies to handle and analyze massive cortical recordings. This being said, prospective therapeutical application for such cBMIs can already be foreseen, as will be described below.

## NEUROFEEDBACK AND COGNITIVE CONTROL

The capacity of the brain to restore and ameliorate its functioning after a major trauma or disease is still poorly understood. It is known that after a CNS disease or trauma (such as a stroke), the brain undergoes extensive functional reorganization (Murase et al., 2004; Nudo, 2006; He et al., 2007; Grefkes et al., 2008; Wang et al., 2010). Building on these impressive adaptive capabilities of the brain, researchers have, in the last 50 years, investigated the ability of the brain to modulate its activity and improve overt behavioral performance thanks to neurofeedback techniques. Initially, this technique consisted in continuously providing the patient with a feedback on the level of activity of a specific cortical region (e.g., thanks to an auditory or visual feedback correlating with the intensity of this cortical activity) and instructing the patient to increase or decrease this activity by their own volition. It has been proven efficient in treating patients with attention disorders and in reducing seizures in epileptic patients (reviewed below). More recent feedback techniques are not based on the raw cortical signals but rather use decoding procedures as described above in order to quantify the exact information of interest contained in the neuronal signals and provide this information as a feedback to the subjects. The subject’s goal is then to improve this specific information through cognitive control. This is a very promising tool that could be used to target specific functions in order to enhance the activity in the brain, both in patients with cognitive deficits arising from acute brain damage or neurodegenerative or neurodevelopmental conditions, as well as in normal subjects seeking to enhance their own cognitive functions.

### NEUROFEEDBACK AND COVERT NEURONAL ACTIVITY

When we are trained on a specific task, our performance often becomes better. Several studies have investigated the underlying neural bases that account for this behavioral improvement. In visual perceptual tasks that involve difficult discriminations, it has been observed that a behavioral improvement in perceptual sensitivity is strongly coupled with improved neural sensitivity in early and intermediate visual areas (Schoups et al., 2001; Yang and Maunsell, 2004; Hua et al., 2010) as well as in higher visual decision areas (Law and Gold, 2008). The idea behind neurofeedback is precisely grounded on such observations. Indeed, if the brain is capable of modulating its activity through learning, why not by voluntary control of the neuronal activity of specific brain areas? Several fMRI studies have approached this question by providing participants with a visual feedback of the level of activity (BOLD signal) in a specific area of the brain and asking them to increase or decrease this level. These studies have all come to the conclusion that brain activity can be regulated and enhanced volitionally by the subject (Weiskopf et al., 2003; deCharms et al., 2004, 2005; Caria et al., 2007; Scharnowski et al., 2014), even when trading the continuous visual feedback for a monetary reward feedback the value of which correlated with the level of activity of the cortical area of interest (Bray et al., 2007). On a neuronal level, electrophysiological studies not only confirm the above results but reveal a remarkable plasticity of individual neurons to modulate their activity under volitional control (Fetz, 1969, 2007; Cerf et al., 2010; Kobayashi et al., 2010; Schafer and Moore, 2011). For example, Fetz performed, in 1969, a visual and auditory feedback experiment where monkeys were rewarded for increasing the activity of newly isolated neurons in the precentral motor cortex. They observe that the activity could be increased with as much as 50–500% above the initial spike rates.

### NEUROFEEDBACK AND OVERT BEHAVIORAL PERFORMANCE

The above studies demonstrate the feasibility of modulating the activity of our own brain by voluntary control. The next question is thus whether this neuronal modulation has an impact on overt behavior? Being able to increase or decrease the activity in the brain is amazing but quite useless if it does not lead to a measurable change in cognitive performance. In the field of EEG neurofeedback, it has actually been known for a long time that a voluntary change in the EEG rhythm, i.e., in the frequency content of the scalp EEG signals, can improve behavior (Wyrwicka and Sterman, 1968; Sterman et al., 1969). For example, Sterman and colleagues highlighted the specific impact of the *sensorimotor rhythm* (SMR: 12–14 Hz) on the capacity to inhibit ongoing behavior. They used neurofeedback to regulate this electrophysiological signature and thereby the frequency of the refractory seizures of a female patient. Specifically, after several months of EEG neurofeedback training to enhance the SMR, the authors noted that the seizures essentially ceased at the same time that a significant increase in the 11–15 Hz frequency band and a corresponding decrease in lower frequencies were observed (Sterman and Friar, 1972). This initial study was followed by a wave of studies describing the clinical benefits of using EEG driven neurofeedback over placebo experimental designs on patients with seizure disorders refractory to pharmacological treatments (Sterman et al., 1974; Kaplan, 1975; Seifert and Lubar, 1975; Kuhlman and Allison, 1977; Kuhlman, 1978; Sterman and Macdonald, 1978; Lantz and Sterman, 1988; Andrews and Schonfeld, 1992; Hansen et al., 1996). A different line of research using the same technique has tried to treat attention disorders such as attention deficit/hyperactivity disorder (ADHD). The first study conducted by Lubar and Shouse (1976), showed that SMR training improved inattentive symptoms in an 11-year old boy with hyperactivity. Further studies confirm that neurofeedback training has a significant effect on reducing hyperactivity or impulsivity symptoms in ADHD (Lubar, 1991; Lubar et al., 1995; Lindén et al., 1996; Thompson and Thompson, 1998; Kaiser and Othmer, 2000). Remarkably, this SMR-driven neurofeedback has further been proven as equally effective as medication (Rossiter and La Vaque, 1995).

Several recent fMRI-based neurofeedback studies show that the operant control of cortical activity can also lead to changes in behavior and to interesting therapeutical applications. For example, Rota et al. (2009) show that the self-regulation of the activity of the right inferior frontal gyrus improves the identification of emotional prosodic intonations. Haller et al. (2010) show that the operant control of the activity of the auditory cortex allows to improve chronic tinnitus, a condition in which subjects perceive more or less constantly an aversive tone or noise, in the absence of any objective external sound source. More recently, Subramanian et al. (2011) show that the clinical motor symptoms of Parkinson disease patients can be improved thanks to neurofeedback driven by the fMRI activity of their supplementary motor area.

In a single-cell recording study in the non-human primates, Schafer and Moore (2011) show that monkeys can learn to modulate the firing rate of individual prefrontal neurons (specifically recorded in the FEF). Importantly, they show that during up-regulation sessions (as compared to down-regulation), an increased firing rate leads to enhanced target discrimination in the receptive field of that neuron. This is a nice demonstration that the voluntary control of FEF neuronal activity is specifically associated with an enhancement of selective spatial attention. It is interesting to compare the finding to Schafer and Moore (2011) in the non-human primate to those obtained by Scharnowski et al. (2012) using fMRI-driven neurofeedback in human subjects. In this study, Scharnowski et al. (2014) demonstrate that the control of the ongoing spontaneous activity as estimated by the BOLD fMRI signal in the visual cortex results in improved visual perception. The authors further show that these observations correlated with increased effective connectivity between the visual cortex and the superior parietal lobe, suggesting that the improved visual perception resulted from enhanced top-down attentional control processes. Top-down attention has been repeatedly shown to modulate the activity of early visual areas (Brefczynski and DeYoe, 1999; Kastner et al., 1999; Li et al., 2008; Gregoriou et al., 2009). The fact that operant control of the activity of visual cortex did not exclusively involve local processes restricted to this cortical region but also involved long-distance and large-scale networks is an indication that the behavioral effects of neurofeedback are maximized by the involvement of the adaptive capabilities of higher level associative cortical regions such as the parietal or the prefrontal cortex.

The above studies all have in common to require the subjects to either increase or decrease the cortical activity being recorded. Cerf et al. (2010) use a concept that is completely different. Instead of just modulating the level of activity, they ask the subjects to actually enhance the information content of the recorded population activity. Specifically, they show that by focusing attention on a concept represented by a target image, neurons in the medial temporal lobe (MTL), an area involved in generating memories of fact and events (Squire and Zola-Morgan, 1991), increase their activity. In this study twelve patients implanted with intracranial electrodes were instructed to manipulate the display of two superimposed images by modulating the firing rate of four MTL units in their brain. The initial visibility of the two images was 50% and the patients were instructed to enhance a target image so as to make it 100% visible. The visibility of the two images was continuously updated via a real-time decoding procedure reflecting the information contained in the spiking activity of these four neurons about either images. This nicely designed experiment is yet limited because the authors only used four units in the decoder which leads to a straightforward interpretation of the decoding performance. Indeed, in this configuration, an increased decoding performance directly translates into an increase in the activity of the unit specifically tuned to the target image and/or decreased activity for the other units. A decoding performance based on the increases or decreases in the information content of hundreds of neurons would result in a much more complex pattern of neuronal changes, possibly more based on functional population synergies than on the mere increase in the activity of selective cells associated with a decrease in the activity of the non-selective cells. Shibata et al. (2011) demonstrate the feasibility of such an experimental design. Participants were given feedback based on the real-time decoding of BOLD fMRI activity. The decoder was configured to discriminate between different angles of a Gabor patch (10°/70°/130°) based on the fMRI BOLD signal recorded from V1 and V2 prior to the neurofeedback procedure. During the neurofeedback sessions, the participants were instructed to enlarge a green circle presented on the screen. For each subject, the size of the green circle was manipulated by how well they could encode a given Gabor patch orientation in their fMRI BOLD activity. The specific angle driving the change in the circle’s size changed from one subject to the other. All participants succeeded in increasing the circle. They were therefore all able to increase the information content related to the assigned specific angle even though the strategies they overtly reported were far from the true workings of the experiment. Importantly, this led to an increased perceptual sensitivity specific to the angle used during feedback in contrast with the two other angles. The results of this experiment are important in several aspects. First, they prove the feasibility of human subjects voluntarily increasing their cortical information content relative to a specific visual feature. Second, they suggest that training the brain to increase its information content directly leads to an improvement in overt behavior. Third, they indicate that the improvements are specific to the exact feature that is being trained, similarly to what can be obtained through perceptual learning.

## FUTURE DIRECTIONS

### COGNITIVE BRAIN–MACHINE INTERFACES FOR COGNITIVE REHABILITATION

As covered in the previous section, neurofeedback applications based on the raw or interpreted (decoded) cognitive information have already been proven efficient for several rehabilitation applications ranging from auditory tinnitus to Parkinson’s disease, seizures, ADHD. These applications are progressively infusing off the laboratory patient care protocols. For example, several start-ups are now providing ADHD EEG-based neurofeedback game platforms integrating enriched immersive virtual 3D environment technologies with neurofeedback training. A challenge facing these future directions is constructing neurofeedback environments that are optimally targeted to specific pathologies. In the above discussed examples, Parkinson’s disease clinical symptoms were improved using the activity of the supplementary motor area. ADHD was improved using the sensory motor rhythm involved in overt behavior inhibition. These conditions were thus critically improved by targeting the specific cortical nodes of the dysfunction which have been associated with the overt clinical symptoms. The further development of such rehabilitation methods based on neurofeedback will require a tight interaction between fundamental neuroscience research providing an ever growing understanding of the neural bases of cognition and its deficits and clinical neuroscience research evaluating the impact of specific neurofeedback designs on well identified groups of patients (as defined by clear-cut genotypes, functional deficits or behavioral deficits). For example, while SMR-based neurofeedback has been shown to reduce impulsivity in hyperactive ADHD patients, it is expected that ADHD patients with low hyperactivity symptoms but high inattention symptoms will not benefit by this approach, due to a different functional deficit underlying their symptoms.

### COGNITIVE BRAIN–MACHINE INTERFACES TO PALLIATE FOR A DEFICIT IN COMMUNICATION

Most of these above foreseen applications will rely on non-invasive cBMI designs. However, in the case of extremely severe cognitive deficits, cost–benefit recommendations will be needed to evaluate whether invasive cBMIs are ethically acceptable. Two such conditions come to mind. The first condition is the case of total locked-in patients, who are unable to move any muscle of their body including their eyes, while they are otherwise aware and awake. The motor recovery is extremely rare and often very minimal. In a recent report, a locked-in patient was able to communicate via sniffing (Plotkin et al., 2010). Using a direct brain-interface, another total locked-in patient was able to answer yes-or-no questions (Parker, 2003; Keiper, 2006).The second condition that could justify invasive cBMIs corresponds to minimally conscious patients. Unlike patients in a persistent vegetative state, these patients have partially preserved conscious awareness. Recent studies indicate that the overall brain metabolism of these patients is 20–40% lower than that of normal subjects, though slightly higher than that of patients in a vegetative state (Schiff et al., 2005). In addition, several studies indicate some degree of preserved cognitive functions. For example, sounds result in a more widespread activation of the primary auditory and prefrontal associative areas in minimally conscious patients than in vegetative state patients (Laureys et al., 2004), more so when narratives were presented as compared to meaningless narratives played backward (Schiff et al., 2002; Coleman et al., 2007). More recently, and in tight relation with the attention-driven cBMIs discussed above, preserved exogenous attention functions and preserved underlying brain processes have been described in these patients, in association with a marked deficit in endogenous attention processes (Chennu et al., 2013). From a therapeutical perspective, deep brain thalamic stimulation has been described to improve the condition of minimally conscious patients (Laureys et al., 2007). Invasive cBMIs are also potentially interesting in this respect. In a first step, cBMIs can serve to assess and quantify the information content of the baseline or stimulus-induced cortical activity of these patients, and possibly serve to interpret part of their phenomenological experience (pain, surprise, attention, etc.). In a second step, cBMIs associated with focal stimulation approaches such as electric or optogenetic stimulations can help increase the information content of specific cortical regions. As soon as consciousness is high enough for the subject to express a preference (e.g., hearing the name of her loved ones instead of the names of strangers), this activity can be used for feedback-cBMI designs that can further help reinforce the weak yet meaningful endogenous cortical activities of minimally conscious patients. While this can seem like science fiction, all the theoretical and experimental grounds are set to make this possible.

### OPEN FIELD COGNITIVE BRAIN–MACHINE INTERFACES FOR ENHANCED COGNITION

In addition to rehabilitation, there is a growing social pressure for healthy individuals to increase their cognitive performance or preserve it from aging. Several tools are already being used to this goal, ranging from cognitive training (through a growing range of enriched video game applications, see for example Cardoso-Leite and Bavelier, 2014), to cognitive pharmacological enhancers (i.e., drugs primarily developed to treat people with cognitive or motor function difficulties that are used by healthy subjects to improve memory, attention, concentration, and planning, see for example Greely et al., 2008), to off-the-laboratory brain stimulation (transcranial direct current stimulation – tDCS-kits are now commercially available). In this context, given its consequences on behavioral performance as described above, non-invasive neurofeedback applications can be considered as a safe improved alternative to cognitive training, as compared to cognitive enhancers or brain stimulation.

### SHARED COGNITION

In a recent report, Pais-Vieira et al. (2013) describe an astounding brain-to-brain interface (BTBI). The cortical activity of an “encoder” rat, performing a learned sensorimotor task was injected, using intracortical microstimulations, into the matching cortical area of a “decoder” rat that was able to learn to use these alien activity patterns to perform the sensorimotor choices as the “encoder” rat. This opens amazing perspectives. From a rehabilitation point of view, one can think of injecting in target cortical regions of the brain of a patient suffering from a severe cognitive deficit the activity patterns recorded from healthy subjects in well-defined contexts. This is not very different from deep brain stimulation procedures now classically used in Parkinson Disease patients for example or from trans-cranial direct current stimulation applied in severe refractory depression, except that the stimulations would in this case correspond to the cognitive information content of healthy subjects.

### CLOSING THE LOOP

Overall, this review brings together several studies that not only demonstrate the feasibility of decoding spatial attention in real time using a diversity of experimental set-ups, but that also show that this real-time decoding can further be used for rehabilitation purposes. As a concluding note, we would like to highlight the fact that this field of cBMIs, and specifically attention-driven BMIs, is still young and that the reviewed studies mostly represent proofs of concept. We believe that the real-time access to spatial attention signals (and other cognitive information) also has the potential to bring about a novel understanding of the neural bases of these cognitive processes that cannot be accessed by more classical investigation methods. Taking this fundamental neuroscience perspective on cBMI research will also provide a better understanding of why and how neurofeedback improves cognition. These are the crucial challenges the field will need to face in the coming years.

## Conflict of Interest Statement

The authors declare that the research was conducted in the absence of any commercial or financial relationships that could be construed as a potential conflict of interest.
